# In-Situ Damage Evaluation of Pure Ice under High Rate Compressive Loading

**DOI:** 10.3390/ma12081236

**Published:** 2019-04-15

**Authors:** Matti Isakov, Janin Lange, Sebastian Kilchert, Michael May

**Affiliations:** Fraunhofer Institute for High-Speed Dynamics, Ernst-Mach-Institut, Ernst-Zermelo-Straße 4, 79104 Freiburg, Germany; matti.isakov@tuni.fi (M.I.); janin.lange@gmx.de (J.L.); sebastian.kilchert@emi.fraunhofer.de (S.K.)

**Keywords:** ice, high rate loading, compressive loading, Split Hopkinson bar, in-situ fractography

## Abstract

The initiation and propagation of damage in pure ice specimens under high rate compressive loading at the strain rate range of 100 s^−1^ to 600 s^−1^ was studied by means of Split Hopkinson Pressure Bar measurements with incorporated high-speed videography. The results indicate that local cracks in specimens can form and propagate before the macroscopic stress maximum is reached. The estimated crack velocity was in the range of 500 m/s to 1300 m/s, i.e., lower than, but in similar order of magnitude as the elastic wave speed within ice. This gives reason to suspect that already at this strain rate the specimen is not deforming under perfect force equilibrium when the first cracks initiate and propagate. In addition, in contrast to quasi-static experiments, in the high rate experiments the specimens showed notable residual load carrying capacity after the maximum stress. This was related to dynamic effects in fractured ice particles, which allowed the specimen to carry compressive load even in a highly damaged state.

## 1. Introduction

The impact of solid ice, such as hail, on high velocity load carrying structures, such as the leading surfaces of airplanes (wind shields or wing leading edges) may pose a serious threat to safety if the impacting particles are large enough (typically diameter 16 mm to 50 mm) and the relative velocity between them and the moving structure is high enough (>100 m/s) that impact forces are generated which are high enough to cause plastic deformation or damage to the structure [1]. Considerable efforts have been in the past taken to quantitatively predict these effects based on numerical simulations (see for example references [2,3]). One of the main questions related to this kind of work is whether ice exhibits strain rate sensitivity at high rates of loading. There are published reports [4,5,6,7,8] on the measurement of the high strain rate properties of ice using the Split Hopkinson Bar method, which is a well-established technique for the measurement of material responses in the strain rate region of 100 s^−1^ to 10,000 s^−1^ [9]. These previous works have concentrated on measuring the strain rate sensitivity of the maximum strength of ice and the analysis has been mainly carried out based on the measured macroscopic stress-strain curves.

A question which arises is how the measured high strain rate stress-strain curves relate to the damage process within the material. Recent work by Lian et al. [10] carried out with a servo-hydraulic testing machine at strain rates of 2 s^−1^ to 40 s^−1^ indicate that specimen cracking starts already before the maximum stress is reached. Ice is known to be a brittle and stochastic material, exhibiting a large scatter in strength even at quasi-static loading rates [11]. It is thus reasonable to assume that the initiation of cracking in the specimen is a stochastic process both in terms of the required stress and the location of the first crack (s). Furthermore, cracks in ice can travel at velocities in the order of 1000 m/s [10], i.e., at velocities which are relevant in the time-scale of the measurements carried out at high rates of loading. Therefore, it seems that for the correct interpretation of the high strain rate stress-strain curves, the evolving damage within the ice specimens should be followed in-situ during the experiments.

Due to the stochastic nature of ice which associated with the growth of ice crystals, this paper does not claim to provide discrete numbers for the strength of ice. Instead, this paper presents a novel consistent testing methodology for assessing rate effects related to fracture phenomena in ice using a Split Hopkinson Pressure Bar setup instrumented with in-situ high speed imaging. The obtained high resolution images clearly show that localized damage may take place in the specimen shortly after the start of loading and that extensive damage already rapidly propagates in the material before the maximum macroscopic stress is measured. The paper concludes with a discussion of the possible ramifications of these phenomena. 

## 2. Experimental Methods 

### 2.1. Preparation of Specimens

The ice specimens were prepared from demineralized water with the following procedure: First blocks with approximate dimensions of 400 mm × 300 mm × 50 mm were produced by letting the water freeze at −10 °C for 24 h to 48 h. This combination of a relatively high temperature and long freezing time was selected in order to facilitate the escape of any trapped air during the freezing process. The freezing was carried out in an isolating container, which effectively allowed heat flow in only one direction, i.e., the thickness direction of the block. This orientation was maintained throughout the following preparation steps so that in the final specimens the loading axis and the direction of the heat flow during freezing were coincident. After the desired block thickness was achieved, the block was removed from the container and stored at −10 °C for a maximum of one week. The grain structure of the ice was not assessed. However, the specimens are thought to be polycrystalline as this is the more natural way to grow than single crystal ice.

**Figure 1:**
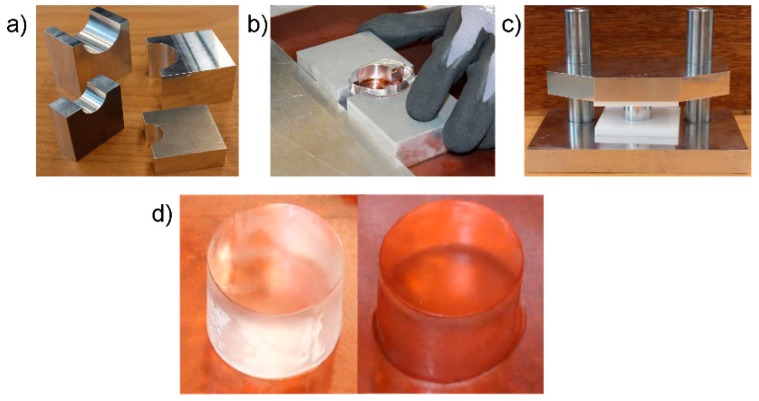
(**a**,**b**) aluminum forms used in the forming of the specimen; (**c**) the jig used to ensure the parallelism of the specimen loading surfaces (the aluminum cylinder at the center illustrates the specimen); (**d**) examples of specimens ready for testing.

In the next step the blocks were mechanically cut to rectangular pieces of approximately 100 mm × 100 mm × 50 mm. These pieces were then carefully shaped to cylinders by using aluminum forms (temperature between +5 °C and +15 °C). After this step the specimens were still larger than the target dimensions, i.e., ~Ø40 mm × 35 mm. Then the specimens were cooled again for 10 min in order to prevent excessive heating of the specimens. After this the specimens were shaped to final dimensions of Ø22 mm × 20 mm by using the aluminum forms shown by Figure 1a,b. The parallelism of the specimen loading surfaces was controlled with a purpose made jig shown in Figure 1c. The final specimens were stored for a maximum of 24 h at the respective test temperature of −20 °C before testing (Figure 1d). In a part of the test series the pre-shaped cylinders were stored for 12 h at −10 °C before final shaping and subsequent testing. This extra step was not, however, observed to affect the mechanical response of the specimens.

Prior to testing, each specimen was measured with a cooled caliber and inspected for any visible damage or trapped air.

### 2.2. High Strain Rate Testing with the Split Hopkinson Pressure Bar

**Figure 2:**
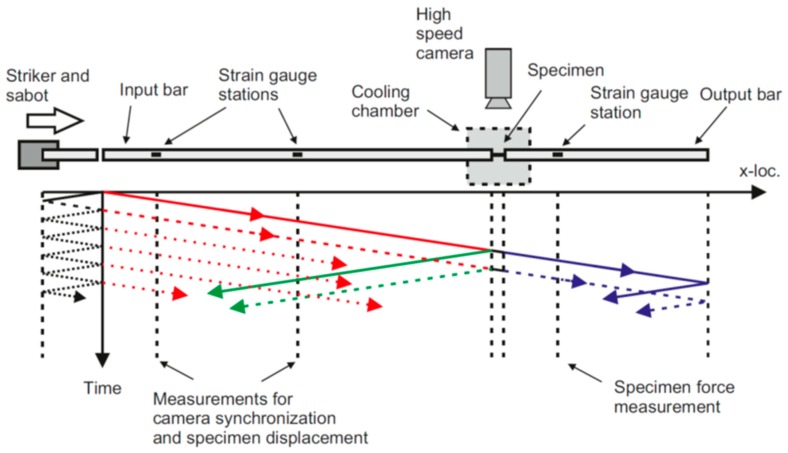
Schematic illustration of the components of the SHPB setup and the relevant wave motion within the bars during the test. The left hand strain gauge on the input bar is referred to as strain gauge 1, the right hand strain gauge on the input bar is referred to as strain gauge 2.

The Split Hopkinson Pressure Bar setup (Freiburg, Germany) used in the tests is schematically illustrated by Figure 2. The setup consists of an input bar (diameter 22 mm, length 2200 mm), output bar (diameter 22 mm, length 600 mm), and a striker (diameter 22 mm, length 310 mm, partially enclosed in a polymer sabot). All bars are made of high strength aluminum alloy. The input and output bars are laterally supported by stanchions, which allow for accurate alignment of the bars. The contacting parts of the stanchions are made of Teflon in order to ensure low-friction contact with the bars. Compressed air is used to accelerate the striker to the desired impact speed. In the current test series the striker impact speed was in the order of 10 m/s. A thick piece of paper was used as a pulse shaper between the striker and the input bar in order to reduce high frequency oscillations in the incident wave and to increase its rise time.

**Figure 3:**
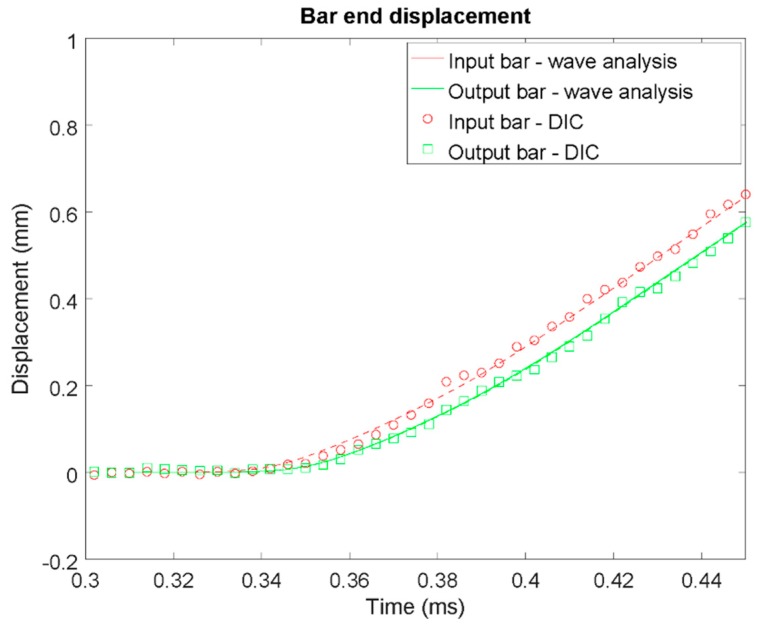
Illustration of the verification of the strain-gauge signal/high speed camera synchronization: bar end displacements obtained from the stress wave analysis and from the high speed footage using digital image correlation. In place of the ice specimens an aluminum cylinder with similar dimensions was used.

The instrumentation used in the tests involved strain gauges attached on two locations in the input bar in order to accurately characterize the wave motion during the test. On the output bar both traditional resistive strain gauges as well as semiconductor strain gauges (for improved measurement sensitivity) were attached on one location on the bar. The strain gauge signals were measured at 10 MHz with a digital oscilloscope. It should be noted that no data filtering was applied on the measurement signals. In addition to the strain gauge signals, specimen deformation and damage was monitored using a high speed camera (Phantom v1610, Wayne, NJ, USA) recording at a frame rate of 250 kHz, allowing—for the first time—detailed in-situ observation of crack initiation and propagation in dynamically loaded ice specimens. Accurate synchronization between the oscilloscope and the camera was obtained using a trigger/feedback signal-loop. Preliminary tests carried out on an aluminum specimen with dimensions similar to the specimens (length 20 mm, diameter 22 mm) were used to verify the synchronization between the two devices (Figure 3). In order to assess the validity of the wave analysis, a black-and-white speckle pattern was applied to the bar ends and the specimen. The commercial digital image correlation software package GOM Correlate (v17, Braunschweig, Germany) was then used to track the movement of the ends of the input bar and the output bar. A subset size of 0.5 mm × 0.5 mm was used for this analysis. It can be seen that a good correlation is achieved between DIC measurements and wave analyses. 

**Figure 4:**
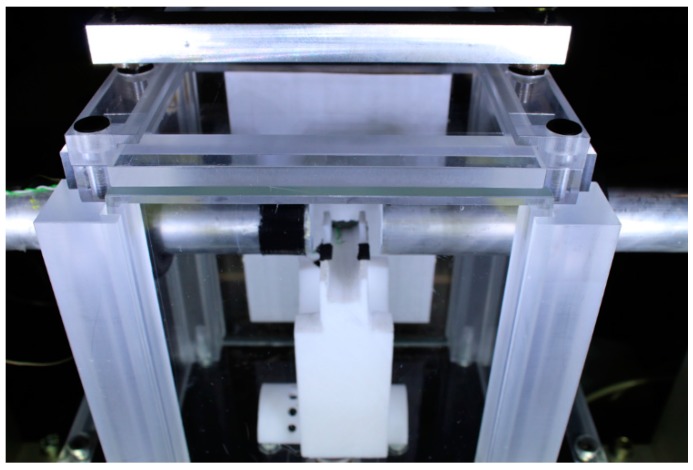
SHPB temperature control chamber made of transparent Plexiglas.

Specimen temperature was controlled by means of a temperature chamber made of transparent Plexiglas, which enclosed the specimen and part of the bars, as shown by Figure 4. A flow of cryogenic nitrogen gas was used to control the chamber temperature. Specimen temperature was verified immediately prior to the impact loading by means of a thermocouple placed in contact with the specimen. In order to ensure low friction contact between the bars and the specimen, Teflon sheets (thickness 0.25 mm) were placed on the contact interfaces. This introduced a challenge in holding the specimen in place before the loading. In this project a specimen support made of Teflon (visible in Figure 4 at the center of the image) was used. The lateral confinement provided by the support column is very limited. The supports are essentially with short line or almost point-contact with the specimen, i.e., there is no cylindrical confinement effect on the specimen which would cause hydrostatic stresses within the specimen. In addition, the relatively low lateral expansion of the test material during compression loading (strain to failure less than 2%) indicates that the effect of lateral confinement is low. This assumption is further justified by the high speed footage presented in Section 3 and Appendix A, which indicates that initial cracking did not take place near the support in any of the tested specimens.

### 2.3. Data analysis of Dynamic Tests on Ice

**Figure 5:**
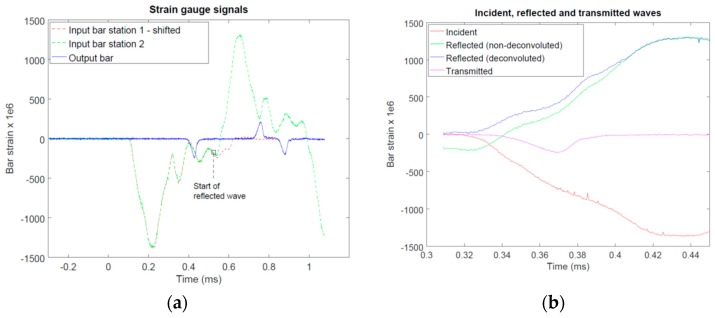
Example of the strain gauge signals recorded during the SHPB tests: (**a**) signals from the strain gauge stations and (**b**) incident, reflected (with and without deconvolution) and transmitted waves after time-shifting to specimen interface location (zoom-in on the early portion of the waves).

In general, the analysis of the SHPB experiments followed the typical practices used in the field [9]. That is, the experiments involved measuring the incident wave generated by the striker into the input bar, the reflected wave originating from the input bar/specimen-interface and traveling in the input bar back towards the striker, as well as the transmitted wave originating from the specimen/output bar-interface and traveling in the output bar in the original direction. The recorded wave data was then analyzed to obtain the force-time and displacement-time response of the specimen based on the theory of uniaxial elastic stress waves in slender bars [9]. In the following section, the key points necessary for the interpretation of the results are presented. As illustrated by Figure 2, the sabot, which partially encloses the striker and remains in contact with the striker throughout the loading, introduces nonperfect release wave generation within the striker. This results in a series of oscillations following the main incident wave, as shown by Figure 5a. Unless corrected for, these trailing incident oscillations partially overlap the reflected wave at the strain gauge station. In order to establish the accurate measurement of the reflected wave, the two strain gauge stations on the input bar were used to separate the trailing oscillations of the incident wave from the reflected wave by means of deconvolution (Equation (1)):

(1) $$\varepsilon_{st2deconv}\left(t\right)=\varepsilon_{st2}\left(t\right)-\varepsilon_{st1}\left(t-\Delta t\right)$$

In Equation (1) *ε_st1_*(*t*) and *ε_st2_*(*t*) refer to the strain signals measured by the first and second strain gauge station on the input bar for time t, respectively. The deconvoluted signal at the second station is denoted by *ε_st2deconv_*(*t*), which also contains the reflected wave signal from the bar-to-specimen interface. The travel time of the longitudinal elastic wave between the two stations is denoted by Δt.

Figure 5b shows an example of the incident, reflected and transmitted wave obtained after carrying out time-shifting to specimen interface location and deconvolution of the reflected wave. Careful analysis of preliminary tests carried out both in the “free end” condition (no contact at the input bar) and in the “bars together condition” (input bar in contact with the output bar without specimen) indicated that the measurements and the deconvolution process carried out for the reflected wave were not accurate enough for the reliable determination of the force acting at the input bar/specimen interface due to the low force carried by the specimen (recall that the force on the input bar is determined as the sum of the incident and reflected waves [9], which makes it highly susceptible to any uncertainty in the measurement especially in the case when the force carried by the specimen is low). Therefore, the longitudinal force acting (*F*) on the specimen was determined only on the specimen/output bar interface (Equation (2)):

(2) $$F\left(t\right)=E_{bar}A_{bar}\varepsilon_{trans}\left(t\right)$$

In Equation (2) *E_bar_* and *A_bar_* denote the Young’s modulus and cross-sectional area of the bar, respectively. The transmitted wave strain amplitude (time-shifted to specimen/output bar interface) is denoted by *ε_trans_*. The accuracy of the measurements and the deconvolution was determined to be sufficient for the calculation of the bar end velocities (*v_input_* and *v_output_*) (Equations (3) and (4)):

(3) $$v_{input}\left(t\right)=-c_{bar}\left(\varepsilon_{inci}\left(t\right)-\varepsilon_{ref}\left(t\right)\right)$$

(4) $$v_{output}\left(t\right)=-c_{bar}\varepsilon_{trans}\left(t\right)$$

In Equations (3) and (4) *ε_inci_*(*t*), *ε_ref_*(*t*), and *ε_trans_*(*t*) denote the incident, reflected (deconvoluted), and transmitted waves moved to the bar/specimen interfaces by time-shifting, respectively. The longitudinal elastic wave speed in the bar is denoted by *c_bar_*. The obtained bar end velocities were integrated over time to obtain displacements and after subtraction, the specimen elongation. The wave analysis was compared with the DIC measurements carried out in a preliminary test with aluminum specimen. As shown in Figure 3, good correlation was obtained between the two measurements.

The high speed video footage of the tests was processed by using the openly available Octave software package (v4.2.1, available at https://www.gnu.org/software/octave/) in the following manner: For each test, the image frame corresponding to the specimen immediately prior to loading was selected as reference. Then each subsequent frame was processed by subtracting the corresponding pixel gray value of the reference frame from the current frame. Finally, the contrast of the processed frame was digitally enhanced by stretching the gray values to correspond to the whole available gray value range. To summarize the data analysis, the measurement data is presented in Section 3 in the following manner: (1) Macroscopic engineering stress-strain response of the specimen calculated based on the force-elongation obtained from the wave analysis and (2) comparison of the stress (force)-time signal obtained from the wave analysis with the processed high speed footage of the specimen cracking pattern. It should be noted that since the effect of the compliance of the Teflon sheets between the specimens and the bars was not corrected for, the stiffness of the test material is underestimated by the results presented here. The time and displacement axis in the presented data refer to the actual specimen loading event as obtained from above described wave analysis and synchronization between the digital oscilloscope and the high speed camera. No further adjustments of the time or strain axis were carried out in the analysis of the experimental data, unless otherwise indicated. The target strain rate for the test series was between 200 s^−1^ and 400 s^−1^. As is seen later in the results, the instantaneous macroscopic strain rate of the specimen varies notably during the tests. This is caused by the fact that the specimen deformation and failure already took place during the phase, at which time the incident wave was still increasing in amplitude.

### 2.4. Quasi-Static Tests

In addition to the high rate tests, quasi-static tests were carried out on similar specimens using a ZwickRoell Z250 (Ulm, Germany) electro-mechanical materials testing machine with an incorporated temperature control chamber. Tests were carried out at a constant displacement rate of 0.05 mm/s, resulting in a nominal strain rate of 0.0025 s^−1^. Similarly to the high rate tests, Teflon sheets (thickness 0.25 mm) were placed on the specimen/anvil-contact surfaces. The measurement data included machine load cell and displacement sensor readings collected at 100 Hz as well as video footage at 10 Hz taken with a digital video camera incorporated in the system. Specimen strain was calculated based on the displacement sensor reading. Since the effect of the compliance of the loading frame or the Teflon sheets was not corrected for, the stiffness of the test material is underestimated by the results presented here.

## 3. Results

**Figure 6:**
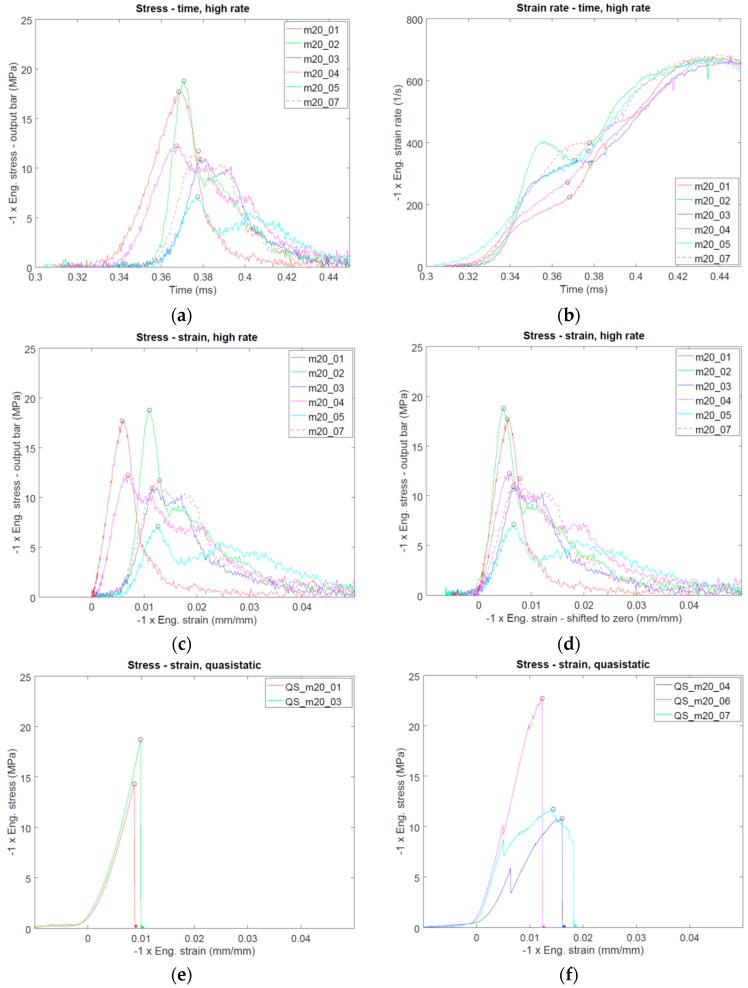
(**a**–**d**): Measured high strain rate data at −20 °C: (**a**) stress-time, (**b**) strain rate-time, (**c**) stress-strain, (**d**) stress-strain with curves shifted to common starting point, (**e**,**f**): measured quasi-static stress-strain data at −20 °C: (**e**) tests with smooth stress-strain curve until maximum stress and (**f**) tests with intermittent drops in stress before maximum stress.

A total of five valid ice tests were performed under quasi-static loading conditions and a total of six valid ice tests were performed under high-rate loading conditions. Figure 6 presents the macroscopic specimen response measured in the high rate tests at −20 °C. As is evident in Figure 6a,c, there is a large scatter in the specimen strength, which reflects the brittle and stochastic nature of the material. As noted above, the instantaneous specimen strain rate varied notably during the tests, as seen in Figure 6b. The strain rate corresponding to the maximum strength was between 200 s^−1^ and 400 s^−1^ in this test series. Despite the scatter in the strength of the specimens, all tests indicate a similar material response: First an almost linear increase of stress with respect to strain until maximum stress and then a gradual decrease in stress with secondary peaks observed in some of the tests. In some tests there is a clear initial zero-stress level in the unshifted stress-strain curve (Figure 6c), which is probably related to a small initial gap between the specimen and the bars (based on the wave analysis, this gap was estimated to be 0.15 mm or less). When the curves are systematically shifted along the strain axis to a common starting point (Figure 6d)), all tests indicate an almost linear initial stress-strain response, though with varying slopes. For quasi-static loading, the measured engineering mean peak stress was 15.6 MPa (COV 31.6%), for high-rate loading, the measured engineering mean peak stress was 12.4 MPa (COV 34.1%). Furthermore, in all high rate tests notable residual load carrying capacity was observed after the maximum stress. 

**Figure 7:**
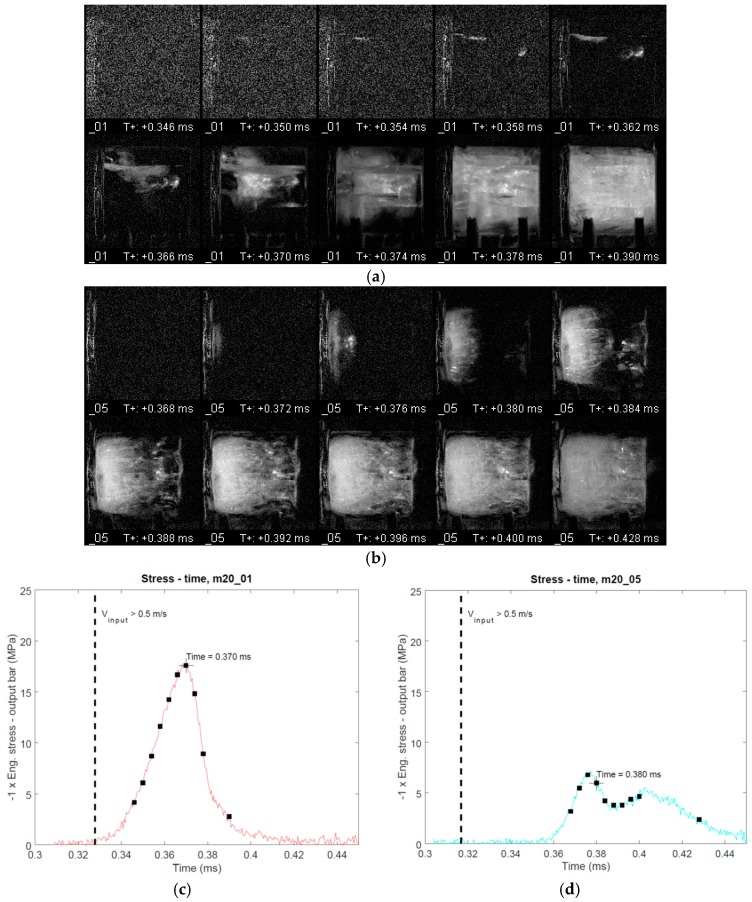
Processed high speed footage obtained in the high rate tests m20_01 (**a**) and m20_05 (**b**) alongside with the corresponding specimen stress–time signal (**c**,**d**) obtained from the output bar strain gauge measurement (synchronized with high speed footage). The input bar is located on the left-hand side of the figure in (**a**,**b**). The time, at which the input bar end velocity exceeded 0.5 m/s as well as the times corresponding to the high speed frames are marked on the respective stress-time curves. The full videos are provided as supplementary material S1 and S2.

**Figure A1:**
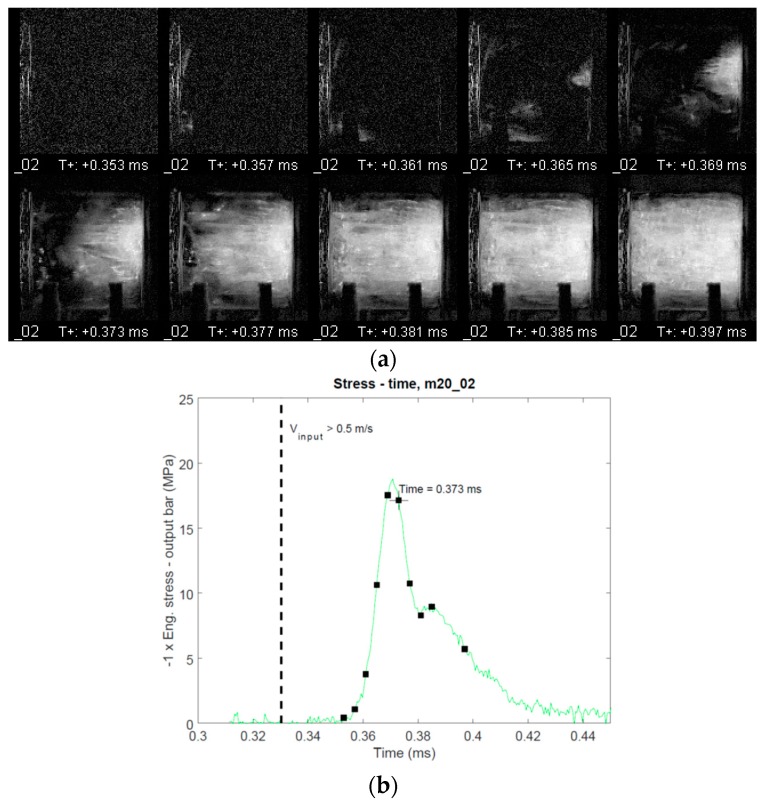
(**a**) processed high speed footage obtained in the high rate test m20_02, (**b**) corresponding specimen stress-time signal obtained from the output bar strain gauge measurement (synchronized with high speed footage). The input bar is located on the left-hand side of the figure in (**a**). The time, at which the input bar end velocity exceeded 0.5 m/s as well as the times corresponding to the high speed frames are marked on the respective stress-time curve.

**Figure A2:**
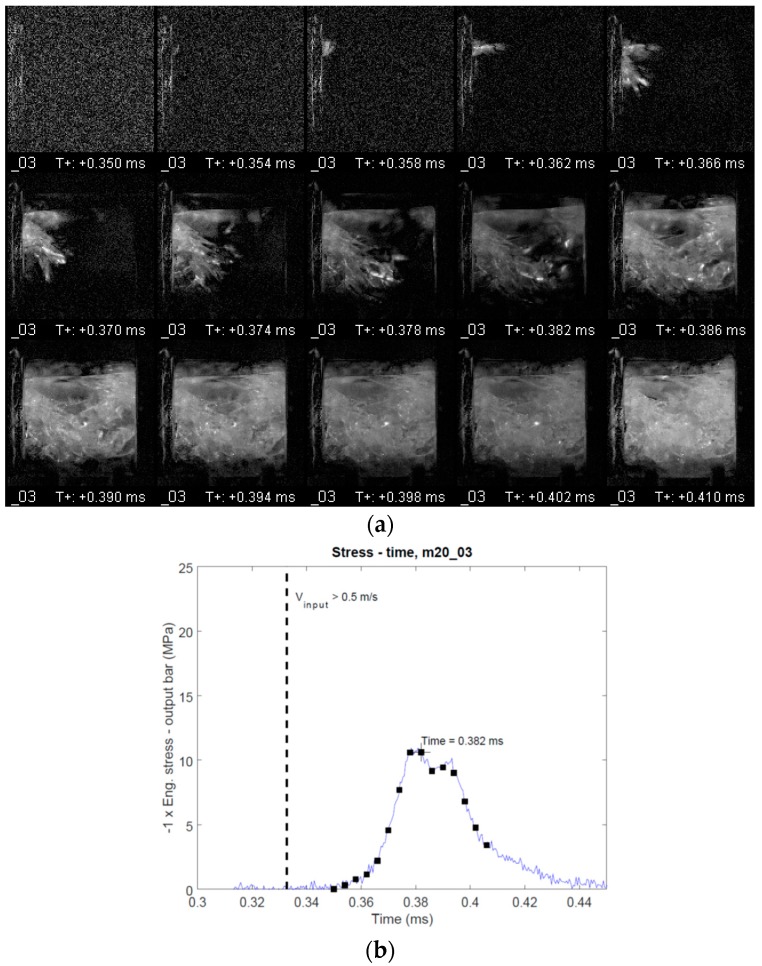
(**a**) Processed high speed footage obtained in the high rate test m20_03, (**b**) corresponding specimen stress-time signal obtained from the output bar strain gauge measurement (synchronized with high speed footage). The input bar is located on the left-hand side of the figure in (**a**). The time, at which the input bar end velocity exceeded 0.5 m/s as well as the times corresponding to the high speed frames are marked on the respective stress-time curve.

**Figure A3:**
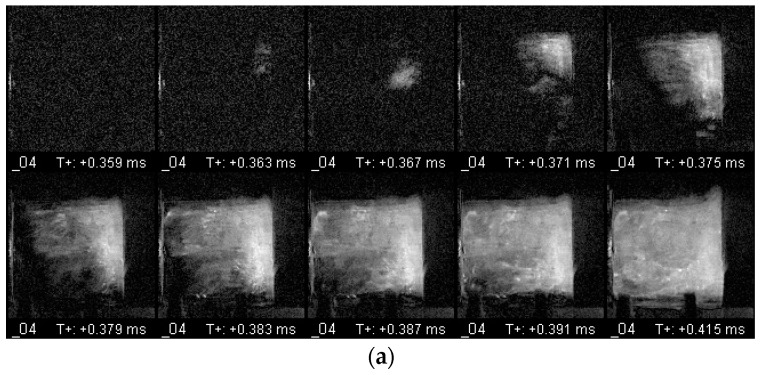

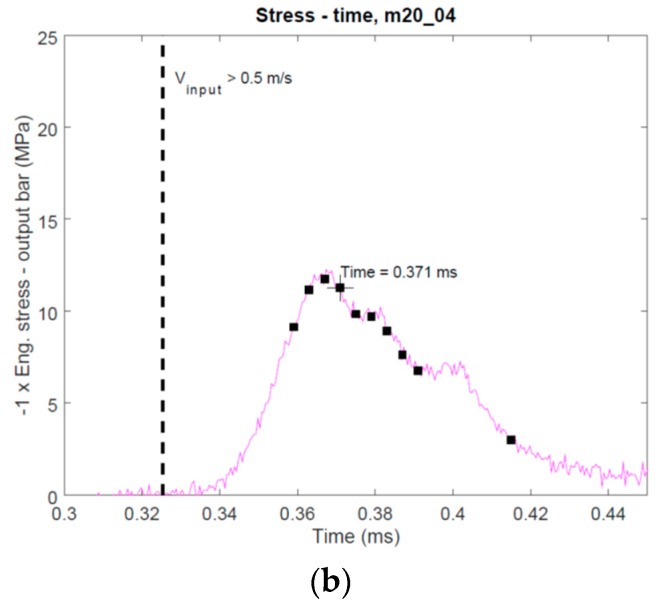
(**a**) processed high speed footage obtained in the high rate test m20_04, (**b**) corresponding specimen stress-time signal obtained from the output bar strain gauge measurement (synchronized with high speed footage). The input bar is located on the left-hand side of the figure in (**a**). The time, at which the input bar end velocity exceeded 0.5 m/s as well as the times corresponding to the high speed frames are marked on the respective stress-time curve.

**Figure A4:**
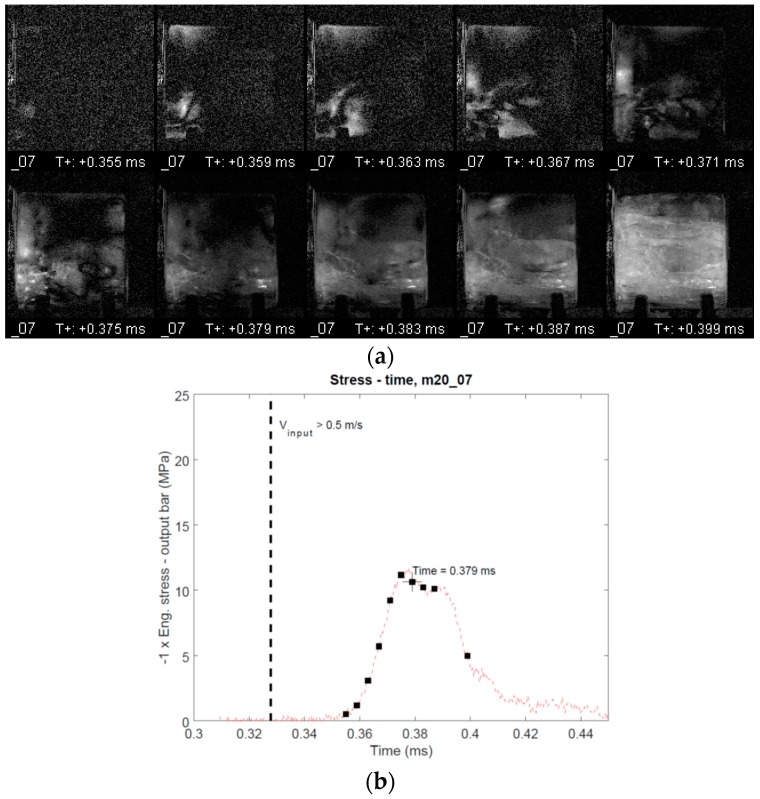
(**a**) processed high speed footage obtained in the high rate test m20_07, (**b**) corresponding specimen stress-time signal obtained from the output bar strain gauge measurement (synchronized with high speed footage). The input bar is located on the left-hand side of the figure in (**a**). The time, at which the input bar end velocity exceeded 0.5 m/s as well as the times corresponding to the high speed frames are marked on the respective stress-time curve.

Figure 7 presents the processed high speed footage for high rate tests m20_01 and m20_05 (please see supplementary material Videos S1 and S2 for the respective videos and Figure A1, Figure A2, Figure A3 and Figure A4 for respective data for the other high rate tests) alongside the synchronized specimen stress signal measured by the output bar strain gauge station. Several points are evident: The damage process does not seem to initiate in any preferred location in the specimens and visible damage already appears before the maximum stress is reached. Furthermore, the maximum stress corresponds relatively well with the time at which damage is seen throughout the specimen length, except for tests m20_04 and m20_05, in which the stress starts to decrease sooner than visible damage is seen throughout the specimen length. In the tests with the highest measured specimen strength, tests m20_01 and m20_02, the initial damage appears to take place as longitudinal cracks along the specimen, especially in test m20_01. In contrast, in tests with lower specimen strength, the damage is more diffuse. This is especially pronounced in the test with the lowest specimen strength, test m20_05, in which a diffuse and wide damage front develops at the input bar/specimen interface and propagates through the specimen volume. In this test the initial zero-stress level in the stress-strain curve (Figure 6c) was the longest, implying that the initial contact between the specimen and the bars was not perfect. This might have contributed to the damage initiating at the input bar side of the specimen. As is evident in the high speed footage, in all high rate tests the damage eventually fills the whole visible specimen volume. Comparison with stress-time data shows that under high rate loading the specimens carry load even in a highly damaged state.

**Figure 8:**
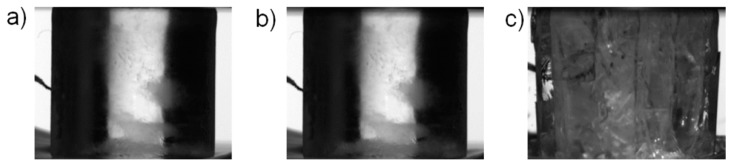
Examples of video frames recorded in the quasi-static test QS_m20_01: (**a**) prior to loading, (**b**) at maximum load, and (**c**) 0.1 s after the previous frame (zero load). The full video is provided as supplementary material S3.

**Figure 9:**
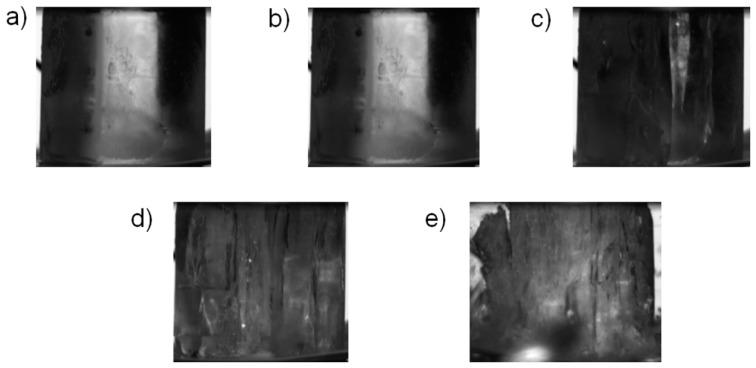
Examples of video frames recorded in the quasi-static test QS_m20_06: (**a**) prior to loading, (**b**) immediately prior to the first drop in load (eng. stress = 10.1 MPa), (**c**) 0.1 s after the previous frame, (**d**) at maximum load (eng. stress 22.7 MPa), and (**e**) 0.1 s after the previous frame (zero load). The full video is provided as supplementary material S4.

**Figure 10:**
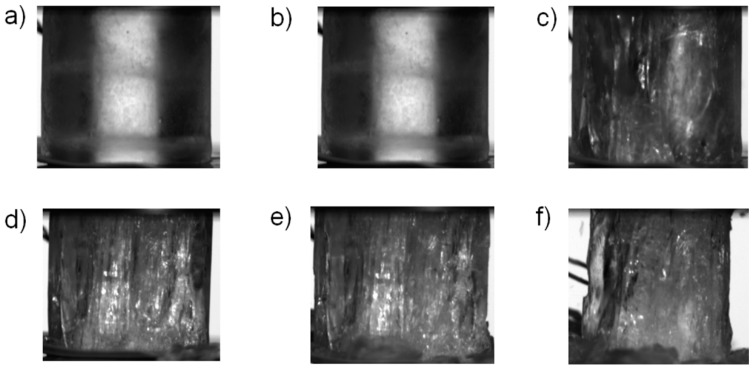
Examples of video frames recorded in the quasi-static test QS_m20_07: (**a**) prior to loading, (**b**) immediately prior to the first drop in load (eng. stress = 8.7 MPa), (**c**) 0.1 s after the previous frame, (**d**) at maximum load (eng. stress = 11.7 MPa), (**e**) after maximum load, immediately prior to the final failure (eng. stress = 8.4 MPa) and (**f**) 0.3 s after the previous frame (zero load). The full video is provided as supplementary material S5.

Figure 6e,f present the results of the quasi-static tests carried out at −20 °C. In these tests brittle behavior after the stress maximum was observed, except for the test QS_m20_07. In tests QS_m20_01 and QS_m20_03 the stress-strain curve was smooth until maximum stress, at which point the specimen failed practically instantaneously (when compared to the time scale of the quasi-static test). The brittle failure was also clearly seen in the recorded video footage, as shown in Figure 8 and in supplementary material S3 for test QS_m20_01. Careful inspection of the quasi-static data (Figure 6f) reveals that in some of these tests (QS_m20_04, QS_m20_06, QS_m20_07) intermittent drops in specimen stress took place before the maximum stress was reached. The occurrence of the load drops could be well related to partial cracking taking place in the specimen, as shown by Figure 9 for test QS_m20_06. The full video is provided as supplementary material S4. In test QS_m20_07, in which the slope of the stress-strain curve decreases notably after the first drop in stress (Figure 6f), continuous formation of longitudinal cracks was observed before and after the point of maximum stress until the final failure took place, as shown in Figure 10. 

## 4. Discussion

As was expected from previous reports (c.f. [11]), the strength of ice showed some scatter, which is typical for brittle materials. However, the macroscopic strength of the tested ice is not notably loading rate sensitive in the studied cases. In contrast, the data indicates a clear loading rate dependence in the damage and failure behavior of the specimens. In quasi-static tests specimen failure was observed to take place either by brittle-like immediate cracking at the maximum stress or by gradual formation of longitudinal cracks resulting in columns, which carried load until maximum stress was reached. In both cases almost no residual load carrying capacity of the specimen after reaching the maximum stress was observed. In contrast, in high rate tests initial specimen damage was localized and often diffuse in appearance. The point of maximum stress was observed to coincide relatively well with the point at which damage was seen throughout the specimen length. Furthermore, all tested high rate specimens showed notable residual load carrying capacity after the maximum stress similarly to previous studies [4,6,7]. 

Based on the high speed footage it is reasonable to assume that the mechanical behavior observed at high rates is closely related to the crack propagation velocity in ice. Due to the diffuse nature of the observed high rate damage, the velocity of an individual crack is challenging to determine based on the current data. However, an order of-magnitude estimate for the propagation of local damage within the specimen can be obtained by assuming that initial damage forms at one of the contact surfaces of the specimen immediately after the start of loading on the specimen/output bar-interface and that maximum stress is reached when the specimen is damaged throughout its length. This means that the propagation time of the cracks through the specimen was in the same order of magnitude as the overall test duration. With these assumptions and by noting that the time until maximum stress was between 15 μs and 40 μs in the tests, it can be calculated that damage propagates through the specimen (length 20 mm) at an average velocity between 500 m/s and 1300 m/s. Even though this calculation is only a rough estimation, it results in a propagation speed which is in accordance with previous reports. Lian et al. [10] reported a value of 1000 m/s for uniaxial compression tests carried out at the strain rate of 10 s^−1^. Pereira et. al [12] reported a value of ~2400 m/s in impact tests carried out at ~200 m/s on cylindrical specimens, whereas Tippman et al. [3] reported a value of 2000 m/s for spherical impact specimen traveling at 60 m/s. Furthermore, the crack propagation velocity is in the same order of magnitude as the longitudinal elastic wave speed in ice (the continuum properties, density 897.6 kg/m3 and Young’s modulus 9.31 GPa, reported by Carney et al. [2], result in a longitudinal elastic wave speed of 3200 m/s). On the other hand, Smith and Kishoni [13], reported based on ultrasonic measurements elastic wave speeds of 3940 m/s and 1990 m/s for a compressional and a shear wave in ice, respectively. It thus seems that the cracks in ice might be able to propagate close to the elastic shear wave speed.

For the current high strain rate test series the above discussed notion leads to an important conclusion: It is likely that when specimen damage initiates and propagates early on during the loading, a state of full force equilibrium does not necessarily exist in the whole specimen volume, which implies that the stress measured at the output bar interface does not necessarily indicate the stress near the crack: If one assumes a longitudinal elastic wave speed of 3200 m/s, then the aforementioned rise times until maximum stress, 15 μs and 40 μs, result in distances of 48 mm and 128 mm traveled by the longitudinal elastic loading wave, respectively. These distances correspond approximately to 1 and 3 back-and-forth reflections within the 20 mm long test specimen, respectively. This low number of reflections gives reason to suspect that already at this loading rate the specimen may not be in full force equilibrium and that the stress measured at the specimen/output bar-interface does not necessarily indicate the dynamic strength of the material. It seems likely that the measured stress is affected by the elastic unloading waves initiated from the cracks, which form early during the loading and propagate at a high velocity through the specimen volume. Thus, the local stress acting on the material volume, in which a crack is formed, might differ from the one indicated by the measurement at the specimen/output bar –interface.

The second observation of the test series, i.e., the dynamic load carrying capacity of the specimen after maximum stress seems to be explained by the fact that at high rates the specimen still maintains its coherence for a period of time after reaching the maximum stress despite the extensive damage. By noting that in the high rate tests the post-maximum stress period lasted 30 μs to 70 μs, it seems that in the high rate tests the specimen fragments alone are able to hold them together. Thus, the input bar, which is moving at a velocity of ~10 m/s, imparts a compressive load through the fragmented specimen to the output bar. In contrast, in quasi-static loading the maximum stress corresponds to the point at which the specimen loses its coherence and ability to carry quasi-static load. A more detailed analysis of the residual loading capacity would, however, involve incorporating the possible interaction between the particles, such as the presence of liquid water suggested by Wu and Prakash [7], and is considered beyond the scope of the current study.

## 5. Conclusions

In this work the effect of loading rate on the damage and fracture behavior of pure ice at −20 °C under uniaxial compression was studied. Whilst the ice may not be representative for naturally grown hail, all specimens have been produced with the same methodology, thus allowing qualitative and quantitative assessments within the batch. High rate tests were carried out with the Split Hopkinson Pressure Bar technique in the strain rate region of 100 s^−1^ to 600 s^−1^. Specimen damage and fracture was studied in-situ using high speed imaging. Based on the results the following conclusions can be drawn:

- Under high rate loading damage may initiate and propagate in the specimen even before the peak load is reached. Catastrophic damage only occurs after the peak load was reached.
- Under high rate loading the damage was observed to propagate in the specimen with a rate, which is in the same order of magnitude as the velocity of the elastic loading wave in ice.
- The above-mentioned findings lead to the conclusion that a state of full force equilibrium is not ensured in the specimen when damage initiates and propagates. This finding implies that already at this loading rate the determination of the strength of ice is affected by wave propagation effects. The force equilibrium can theoretically be improved by replacing the aluminum bars with bars matching the impedance of ice more closely. Amongst practical engineering materials, the next option after aluminum would be a technical polymer. However, this approach is believed to bring about more problems than solutions in the current case: The bars would be deforming visco-elastically, thus demanding tedious numerical techniques and experimental calibration in order to accurately interpret the strain gauge signals in the presence of notable dispersion and frequency-dependent damping in the wave motion. In the current study this would be exceptionally challenging due to the short duration of the tests and brittle response of the specimen, which would inevitably lead to notable distortion of the stress waves, as they travel in visco-elastic bars. Furthermore, the mechanical properties of the visco-elastic polymer bars would be most likely affected, when they are subjected to sub-zero temperature in the specimen cooling chamber.
- The fractured specimen can carry notable load when it is compressed at 10 m/s, but not when it is compressed at 0.05 mm/s. This can be explained by dynamic effects which do not occur under quasi-static loading conditions which are four orders of magnitude smaller than the high-rate loading conditions achieved using the Hopkinson Bar apparatus.
- For quasi-static loading, the measured engineering mean peak stress was 15.6 MPa (COV 31.6%), for high-rate loading, the measured engineering mean peak stress was 12.4 MPa (COV 34.1%) indicating a decrease of strength with increasing loading rate. However, at the same time, the post failure response changes with loading rate such that the post-peak load carrying capability is higher for high loading rates. However, due to the small sample size, this lacks stochastic relevance and should be assessed with more tests.

The results of this study clearly show the importance of using in-situ high speed imaging, when the high rate response of ice is studied. A variety of initial cracking morphologies was observed. By using only macroscopic stress-strain curves without in-situ footage, these differences in specimen behavior would be very challenging to detect. This fact has clear implications for example in the development and calibration of high strain rate material models for ice, whose accuracy in many cases relies on the correct description of the fracture propagation within the material.

## Supplementary Materials

The following are available online at https://zenodo.org/record/2649030#.XL7aX5MRWUk, Video S1: Video recording of Hopkinson Bar test HR_m20_01 (input bar on the left, output bar on the right), Video S2: Video recording of Hopkinson Bar test HR_m20_05 (input bar on the left, output bar on the right), Video S3: Video recording of quasi-static test QS_m20_01. Video S4: Video recording of quasi-static test QS_m20_06. Video S5: Video recording of quasi-static test QS_m20_07.

## Appendix A

This appendix shows additional high-speed footage recorded during SHPB testing.
